# Clinical characteristics and outcome of hemodialysis patients with COVID-19: a large cohort study in a single Chinese center

**DOI:** 10.1080/0886022X.2020.1816179

**Published:** 2020-09-14

**Authors:** Rong Zou, Fang Chen, Dan Chen, Cui-Ling Xu, Fei Xiong

**Affiliations:** Institutes Renal Division, Department of Medicine, Wuhan Integrated TCM & Western Medicine Hospital, Tongji Medical College, Huazhong University of Science and Technology, Wuhan, China

**Keywords:** Novel coronavirus disease, COVID-19, hemodialysis, outcome

## Abstract

**Background:**

Novel coronavirus disease (COVID-19) is spreading rapidly, which poses great challenges to patients on maintenance hemodialysis. Here we report the clinical features of 66 hemodialysis patients with laboratory confirmed COVID-19 infection.

**Design, setting, participants, and measurements:**

Retrospective, single-center case series of the 66 hemodialysis patients with confirmed COVID-19 from 1 January to 5 March 2020; the final date of follow-up was 25 March 2020.

**Results:**

The clinical data were collected from 66 hemodialysis patients with confirmed COVID-19. The incidence of COVID-19 in our center was 11.0% (66/602), of which 18 patients died. According to different prognosis, hemodialysis patients with COVID-19 were divided into the survival and death group. A higher incidence of fever and dyspnea was found in the death group compared with the survival group. Meanwhile, patients in the death group were often accompanied by higher white blood cell count, prolonged PT time, increased D-dimer (*p* < .05). More patients in the death group showed hepatocytes and cardiomyocytes damage. Furthermore, logistic regression analysis suggested that fever, dyspnea, and elevated D-dimer were independent risk factors for death in hemodialysis patients with COVID-19 (OR, 1.077; 95% CI, 1.014 to 1.439; *p* = .044; OR, 1.146; 95% CI, 1.026 to 1.875; *p* = .034, OR, 4.974; 95% CI, 3.315 to 6.263; *p* = .007, respectively).

**Conclusions:**

The potential risk factors of fever, dyspnea, and elevated D-dimer could help clinicians to identify hemodialysis patients with poor prognosis at an early stage of COVID-19 infection.

## Introduction

By March 2020, there was a global outbreak of novel coronavirus disease (formerly known as 2019-nCoV and renamed COVID-19) [1]. It affected more than 770,000 people in over 100 countries, including China, Korea, Italy, Iran, Japan, Germany, France, Spain, the United States [2]. From December 2019 to March 2020, 80566 cases of the COVID-19, including more than 3000 deaths, had been confirmed in China [3].

As the center of the novel coronavirus disease outbreak in 2019, Wuhan has more than 4000 maintenance hemodialysis patients. Because of the older age, multiple comorbid conditions and suppressed immune systems, there is an increased risk of novel coronavirus infection in these hemodialysis patients. It is reported that the mortality rate of novel coronavirus disease has been approximately 2%, which is lower than severe acute respiratory syndrome (SARS; mortality rate, >40% in patients aged >60 years) and the Middle East respiratory syndrome (MERS; mortality rate, 30%) [4,5]. However, little is known about clinical characteristics of hemodialysis patients with COVID-19. Here we report the clinical features and outcome of 66 hemodialysis patients with COVID-19 from a single hemodialysis center.

## Methods

A retrospective study was conducted focusing on the clinical characteristics of confirmed cases of COVID-19 in hemodialysis center of Wuhan Integrated TCM & Western Medicine Hospital. All hemodialysis patients with COVID-19 were recruited From January 1 to March 5, 2020. Novel coronavirus disease (COVID-19) was defined according to the WHO interim guidance [6]. All confirmed patients were diagnosed by symptoms and radiological findings plus positive laboratory results. A confirmed case with COVID-19 was defined as a positive result to high-throughput sequencing or real-time reverse-transcriptase polymerase-chain-reaction (RT-PCR) assay for nasal and pharyngeal swab specimens. Only the laboratory-confirmed cases were included in the analysis. Epidemiological data, symptoms at presentation, presence of comorbidities, laboratory data, treatment regimens, and clinical outcomes were collected from electronic medical records using a standardized data collection form. The frequency of examinations was determined by the treating physician. All recruited patients received the computed tomography scan. Hemodialysis patients with COVID-19 were followed up to March 25, 2020. All data were checked by two physicians (CF and CD) and a third researcher (XF) adjudicated any difference in interpretation between the two primary reviewers. This study was approved by the ethics commissions of Wuhan Integrated TCM & Western Medicine Hospital (ID number: MEC[2020]No.8) and informed consent was obtained from patients involved before enrollment.

### Laboratory confirmation

Throat swab samples were collected and sent to the laboratory in specialized hospitals for novel coronavirus-infected hemodialysis patients. The laboratory diagnosis of COVID-19 was implemented by the Chinese Center for Disease Control and Prevention before January 23, 2020, and then by a certified tertiary hospital. The RT-PCR assay was conducted by the protocol established by the World Health Organization [7].

### Chest CT severity score assessment

Chest CT imaging was performed on a 16-detector CT scanner (Emotion; SIEMENS). CT images were then acquired in the supine position during a single inspiratory breath-hold. The scanning range was from the apex of lung to costophrenic angle.

The abnormalities of the pulmonary parenchyma were estimated according to the semi-quantitative scoring systems established by Feng Pan and his colleagues [8]. In brief, two chest radiologists who were blinded to patients’ data evaluated all the CT images separately, in a standard clinical Picture Archiving and Diagnostic System workstation. Each of the five lung lobes was visually scored on a scale of 0 to 5(‘0’ indicating 0% involvement; ‘1’ indicating less than 5% involvement; ‘2’ indicating 5%–25% involvement; ‘3’ indicating 26%–49% involvement; ‘4’ indicating 50%–75% involvement; ‘5’ indicating more than 75% involvement). The total CT score was determined as the sum of lung involvement, ranging from 0 (no involvement) to 25 (maximum involvement). If there was a dispute, final scores were determined by consensus.

### Statistical analysis

Results were expressed as mean ± SD (for data that were normally distributed), or median and inter-quartile range (IQR) (for data that were not normally distributed). Differences in quantitative parameters between groups were assessed using the t-test (for data that were normally distributed) or nonparametric tests (for data that were not normally distributed). Differences in semi-quantitative results were tested using the Mann Whitney U test. Differences in qualitative results were compared using the χ2 test. Logistic regression was used to analyze the factors related to death. It was considered a significant difference if the *p*-value was less than .05. The analysis was performed with SPSS statistical software package (version 13.0; Chicago, IL).

## Results

From December 1 to March 5, a total of 66 hemodialysis patients in our center were diagnosed with novel coronavirus infection. Compared with the non-infected hemodialysis patients, patients with COVID-19 had a significantly shorter dialysis age, lower level of plasma albumin, the higher level of blood creatinine, potassium, and parathyroid hormone (all *p* < .05, Table 1). No significant difference in age, gender, primary kidney disease, coexisting disorders, average blood pressure, KT/V, and type of angioaccess was found between the COVID-19 infected patients and non-infected patients (Table 1).

**Table 1.** Baseline characteristics in hemodialysis patients with or without COVID-19.

	non-infected patients *N* = 536	COVID19-infected patients *N* = 66	*p*
Age (yrs, median and IQR)	61 (52.0, 69.0)	64.5 (57.0, 72.0)	.085
Dialysis age ( mon, median and IQR)	69 (47.0, 96.9)	60.0 (37.0, 78.5)	**.004**
Male sex (No.,%)	299/536 (55.8)	31/66 (47.0)	.175
Smoking history (No.,%)	285/536 (53.2)	34/66 (51.5)	.799
Primary disease (No.,%)			.507
Chronic glomerulonephritis	215/536 (40.1 )	24/66 (36.4)	
Hypertensive nephropathy	123/536 ( 22.9)	17/66 (25.8)	
Diabetic nephropathy	98/536 (18.3)	16/66 (24.2)	
Others	100/536 (18.7)	9/66 (13.6)	
Coexisting disorders (No.,%)			.948
Chronic obstructive pulmonary disease	73/536 (13.6)	10/66 (15.2)	
Coronary heart disease	187/536 (34.8)	20/66 (30.3)	
Cerebrovascular diseases	78/536 (14.6)	9/66 (13.6)	
Cancer^a^	28/536 (5.2)	4/66 (6.1)	
Chronic liver disease^b^	26/536 (4.9)	4/66 (6.1)	
Haemoglobin (g/L, mean ± SD)	103.7 ± 14.5	103.6 ± 17.4	.980
Albumin (g/L, mean ± SD)	38.7 ± 3.6	35.2 ± 4.6	**.003**
BUN (mmol/L, mean ± SD)	17.5 ± 8.1	16.3 ± 8.7	.267
Creatinine (μmol/L, mean ± SD)	725.6 ± 297.4	831.7 ± 317.6	**.007**
Phosphorus (mmol/L, median and IQR)	1.7 (1.1, 2.0)	1.8 (0.9, 2.2)	.412
Potassium (mmol/L, median and IQR)	4.6 (4.2, 5.1)	4.8 (4.3, 5.1)	**.007**
Parathyroid hormone (ng/L, median and IQR)	341.6 (198.8, 543.6)	430.9 (220.3, 430.9)	**.007**
Monthly averaged pre-HD systolic Bp (mmHg, mean ± SD)	144 ± 19	138 ± 22	.514
Monthly averaged pre-HD diastolic Bp (mmHg, mean ± SD)	85 ± 12	87 ± 14	.672
Intradialytic drop in systolic BP (mmHg, mean ± SD)	20 ± 10	22 ± 12	.745
KT/V (median and IQR)	1.4 (1.3, 1.4)	1.3 (1.2, 1.4)	.289
Type of angioaccess (No.) Catheter vs graft vs fistula	69/6/461	6/0/60	.388

Data are presented as medians (interquartile ranges, IQR) and n/N (%). All cases were stable disease. *p* Values denoted the comparison between the survival group and death group. *p* < .05 was considered statistically significant (shown in bold).

^a^Cancers referred to any malignancy.

^b^Chronic liver disease referred to hepatitis B and hepatitis C.

In hemodialysis patients with COVID-19, the median age was 64.5 years (IQR 57.0, 72.0 yr), and 35(53.0%) were females. Their median duration of hemodialysis was 60.00 months (IQR 37.0, 78.5 mo, Table 2). The incidence of coronavirus infection in our center was 11.0% (66/602), of which 18 patients died. The mortality rate in the center was 27.3% (18/66), which was much higher than the general population, even in Wuhan city (nearly 5.1%, 2574/50008). Similar to the general population, Cough (69.7%) and fever (37.9%) were still the most common symptoms in hemodialysis patients with COVID-19, whereas nasal congestion (6.1%) was rare. Other symptoms were fatigue (34.8%), dyspnea (16.7%), sputum production (10.6%), diarrhea (7.6%), nausea and vomiting(15.2%), conjunctival congestion (7.6%). Thirty-four (51.5%) of the 66 patients had a smoking history. Fifty-six (84.8%) of the 66 patients were transferred to a specialized hospital after diagnosis, as well as 8(12.1%) patients into a mobile cabin hospital. Only two hemodialysis patients with confirmed COVID-19 refused hospitalization and treated in the emergency department. Ten of the 66 patients (15.2%) had chronic obstructive pulmonary disease, 20(30.3%) had coronary heart disease, 9(13.6%) had cerebrovascular diseases, 4(6.1%) had cancer, and 4(6.1%) had chronic liver disease (Table 2).

**Table 2.** Clinical features of hemodialysis patients with COVID-19.

	All COVID19- infected patients *N* = 66	Survival Group *N* = 48	Death Group *N* = 18	*p*
Age (years, median and IQR)	64.5 (57.0, 72.0)	65.5 (57.0, 70.5)	60 (52.0, 73.0)	.629
Dialysis age ( mon, median and IQR)	60.0 (37.0, 78.5)	60.5 (38.0, 72.0)	54.0 (26.0,84.0)	.194
Male sex (No.,%)	31/66 (47.0)	20/48 (41.7)	11/18 (61.1)	.178
Smoking history (No.,%)	34/66 (51.5)	25/48 (52.1)	9/18 (50.0)	1.000
Hospitalization (No.,%)	56/66 (84.8)	40/48 (83.3)	16/18 (88.9)	.715
Time from the initial diagnosis to hospitalization (days, median and IQR)	2.0 (2.0 3.8)	2.0 (2.0, 3.8)	2.0 (2.0, 3.5)	.961
Time from onset of symptoms to hospitalization (days, median and IQR)	8.0 (6.0, 11.0)	8.0 (5.0, 12.0)	7.6 (6.0, 11.0)	.647
Primary disease (No.,%)				
Chronic glomerulonephritis	24/66 (36.4)	19/48 (39.6)	5/18 (27.8)	.168
Hypertensive nephropathy	17/66 (25.8)	14/48 (29.2)	3/18 (16.7)	
Diabetic nephropathy	16/66 (24.2)	11/48 (22.9)	5/18 (27.8)	
Others	9/66 (13.6)	4/48 (8.3)	5/18 (27.8)	
Coexisting disorders (No.,%)				
Chronic obstructive pulmonary disease	10/66 (15.2)	7/48 (14.6)	3/18 (16.7)	1.000
Coronary heart disease	20/66 (30.3)	10/48 (20.8)	10/18 (55.6)	**.014**
Cerebrovascular diseases	9/66 (13.6)	6/48 (12.5)	3/18 (16.7)	.696
Cancer^a^	4/66 (6.1)	2/48 (4.2)	2/18 (11.1)	.298
Chronic liver disease^b^	4/66 (6.1)	2/48 (4.2)	2/18 (11.1)	.298
Monthly averaged pre-HD systolic Bp (mmHg, mean ± SD)	140 ± 24	142 ± 24	140 ± 22	.724
Monthly averaged pre-HD diastolic Bp (mmHg, mean ± SD)	87 ± 19	87 ± 18	89 ± 16	.582
intradialytic drop in BP (mmHg, mean ± SD)	20 ± 12	20 ± 10	22 ± 12	.720
KT/V (median and IQR)	1.3 (1.2, 1.4)	1.4 (1.2, 1.4)	1.3 (1.2, 1.4)	.686
type of angioaccess (No.) catheter vs graft vs fistula	6/0/60	4/0/44	2/0/16	1.000
symptoms (No.,%)				
Fever (No.,%)	25/66 (37.9)	11/48 (22.9)	14/18 (77.8)	**.000**
Cough (No.,%)	46/66 (69.7)	30/48 (62.5)	16/18 (88.9)	.069
Sputum production (No.,%)	7/66 (10.6)	4/48 (8.3)	3/18 (16.7)	.327
Diarrhea (No.,%)	5/66 (7.6)	3/48 (6.3)	2/18 (11.1)	.608
Nausea and vomiting (No.,%)	10/66 (15.2)	7/48 (14.6)	3/18 (16.7)	1.000
Conjunctival congestion (No.,%)	5/66 (7.6)	3/48 (6.3)	2/18 (11.1)	.608
Nasal congestion (No.,%)	4/66 (6.1)	3/48 (6.25)	1/18 (5.55)	1.000
Fatigue (No.,%)	23/66 (34.8)	15/48 (31.3)	8/18 (44.4)	.388
Dyspnea (No.,%)	11/66 (16.7)	4/48 (8.3)	7/18 (38.9)	**.007**

Data are presented as mean (SD, standard deviation), medians (interquartile ranges, IQR) and n/N (%).

*p* Values denoted the comparison between the survival group and death group. *p* < .05 was considered statistically significant (shown in bold). ^a^Cancers referred to any malignancy. ^b^Chronic liver disease referred to hepatitis B and hepatitis C.

**Figure 1.**
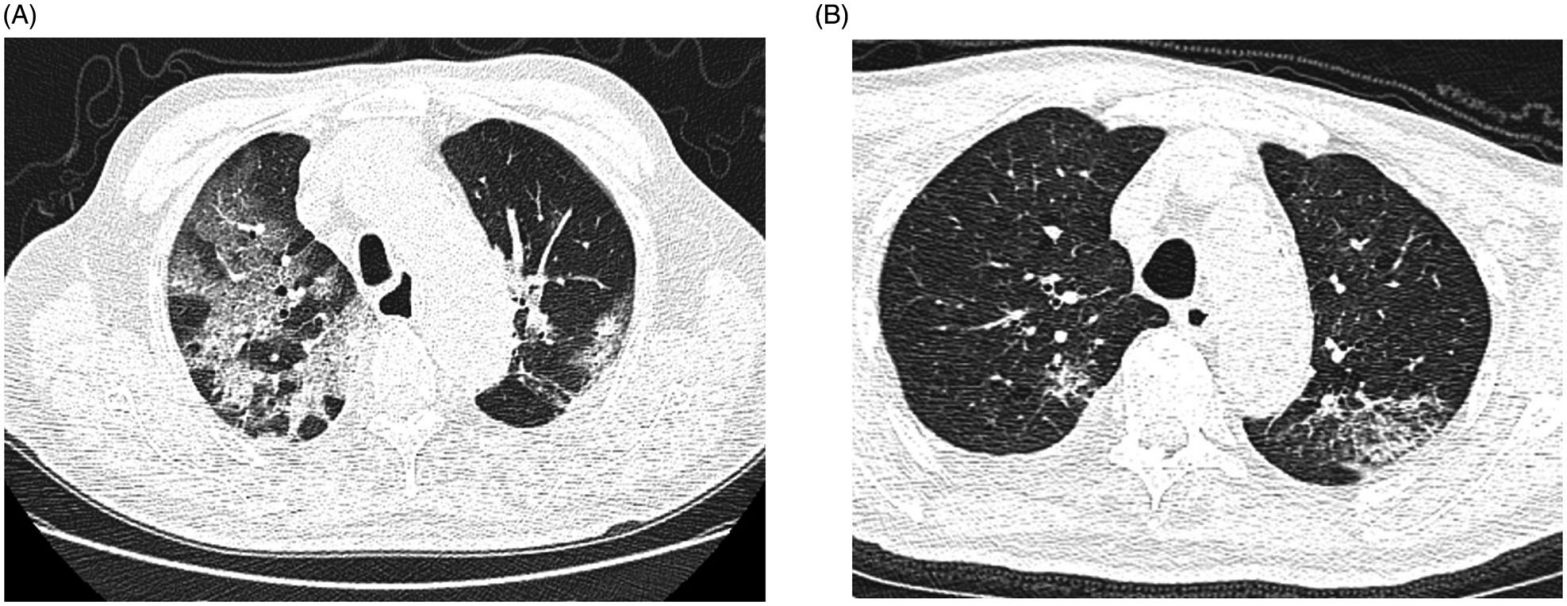
Representative chest CT findings in the death group (A) and survival group (B).

Following the different endpoints, 66 patients were categorized into survival and death subgroups. Compared with the survival group, patients in the death group had a significantly higher incidence of fever and dyspnea (37.9% vs. 22.9%, *p* < .001; 16.7% vs. 8.3%, *p* < .001, respectively, Table 2). Meanwhile, the incidence of coronary heart disease in hemodialysis patients in the death group was higher than that in the survival group(55.6% vs. 20.8%, *p* < .05, Table 2). No significant difference in age, gender, dialysis age, primary kidney disease, average blood pressure, KT/V, and type of angioaccess was found between the two groups (Table 2). However, patients in the death group had more prominent laboratory abnormalities than those in the survival group, such as leukocytosis, lymphopenia, elevated C-reactive protein levels, extended PT time, and elevated D-dimer level (all *p* < .05, Table 3). Meanwhile, hepatocyte damage was significantly more severe in the death group than that in the survival group (i.e. higher level of alanine aminotransferase and aspartate aminotransferase, lower level of albumin, all *p* < .05, Table 3). In terms of cardiomyocyte damage, no significant difference was found in creatine kinase isoenzyme-MB (CK-MB) level between the two groups. However, the level of cardiac troponin-I was higher in the death group than that in the survival group (0.572(IQR 0.020, 0.173) vs. 0.071(IQR 0.063, 0. 730), *p* = .002, Table 3). Further logistic regression analysis suggested that fever, dyspnea, elevated D-dimer level were independent risk factors for death in hemodialysis patients with COVID-19(OR, 1.077; 95% CI, 1.014 to 1.439; *p* = .044; OR, 1.146; 95% CI, 1.026 to 1.875; *p* = .034, OR, 4.974; 95% CI, 3.315 to 6.263; *p* = .007, respectively; Table 4). Almost all hemodialysis patients with COVID-19 had characteristic CT features in the disease process, such as different degrees of ground-glass opacities, multifocal organizing pneumonia, and architectural distortion in a peripheral distribution, but the positive chest CT findings in the death group were more severe than that in the survival group at the beginning of disease (Table 3; Figure 1). According to the semi-quantitative scoring system, the total scores of lung involvement in the death group was greater than that in the survival group ( 6 ± 4 vs. 2 ± 2; *p* = .021) (Table 3).

**Table 3.** Results of laboratory and radiographic investigations in hemodialysis patients with COVID-19.

Laboratory findings	Normal range	Survival group *N* = 48	Death group *N* = 18	*p*
Blood leukocyte count (No.,%)	3.5–9.5			
>10 × 10^9^/L		4/48 (8.3)	6/18 (33.3)	**.020**
4–10^9^/L		23/48 (33.8)	9/18 (42.9)	.450
<4 × 10^9^/L		21/48 (43.8)	3/18 (16.7)	**.049**
Lymphocyte count (No.,%)	1.1–3.2			
< 1.1*10^9^/L		33/48 (68.8)	16/18 (88.9)	.122
Platelet count (10^9^/L, mean ± SD)	125–350	170.2 ± 69.2	141.7 ± 58.6	.155
Haemoglobin (g/L, mean ± SD)	130–175	104.4 ± 19.9	102.3 ± 19.8	.721
Prothrombin time (s, median and IQR)	11.5–14.5	13.7 (12.9, 15.4)	18.8 (12.7, 22.3)	**.017**
Activated partial thromboplastin time (s, median and IQR)	28.0–44.0	39.6 (35.5, 43.0)	42.7 (31.3, 44.9)	.095
D-dimer (mg/L, mean ± SD)	0–1	1.9 ± 0.7	4.2 ± 2.5	**.010**
C-reactive protein (mg/L, median and IQR)	0.0–5.0	28.2 (17.7, 99.4)	78.0 (19.6 131.8)	**.019**
Procalcitonin (μg/L, median and IQR)	0.0–5.0	3.9 (1.6, 6.4)	9.6 (1.0 18.2)	**.000**
Lactose dehydrogenase (IU/L, median and IQR)	313–618	358.0 (357.1, 509.9)	605.5 (491.6, 826.4)	**.002**
Aspartate aminotransferase ( IU/L, median and IQR)	13–69	18.5 (11.5, 33.3)	76.3 (27.7, 168.7)	**.001**
Alanine aminotransferase (IU/L, median and IQR)	15–40	24.1 (15.4, 44.0)	63.4 (21.0, 75.6)	**.009**
Albumin (g/L, mean ± SD)	35–50	38.1 ± 3.8	35.3 ± 5.0	**.047**
Total bilirubin (μmol/L, median and IQR)	3–22	5.9 (4.5, 7.8)	7.9 (4.4, 11.2)	.188
Creatinine (μmol/L, mean ± SD)	71–133	791.9 ± 410.7	895.8 ± 317.4	.365
TCO_2_ (mmol/L, median and IQR)	22.0–30.0	22.6 (21.6, 24.5)	18.4 (15.5, 23.1)	**.031**
Calcium (mmol/L, median and IQR)	2.1–2.5	2.1 (1.8, 2.4)	2.1 (1.9, 2.6)	.852
Phosphorus (mmol/L, median and IQR)	0.81–1.45	1.9 (1.2, 2.2)	1.6 (0.9, 2.1)	.112
Sodium (mmol/L, median and IQR)	137.0–145.0	140.9 (135.7, 142.3)	137.3 (135.8, 140.6)	.069
Potassium (mmol/L, median and IQR)	3.5–5.1	4.6 (4.3, 5.1 )	4.8 (4.0, 5.5)	.947
Parathyroid hormone (ng/L, median and IQR)	15.0–65.0	373.1 (251.9, 655.6)	282.1 (201.8, 555.6)	.118
Left Ventricular Ejection Fractions (No.,%)				.230
< 40%		3/48 (6.3)	2/18 (11.1)	
40-49%		6/48 (12.5)	5/18 (27.8)	
≥50%		31/48 (64.6)	11/18 (50.0)	
cardiac troponin-I (μg/L, median and IQR)	0.000–0.026	0.071 (0.020, 0.173)	0. 572 (0.063, 0. 730)	**.002**
creatine kinase isoenzyme-MB (IU/L, median and IQR)	0–24	10.6 (7.8, 21. 5)	9.5 (8.3, 16.7)	.352
Abnormalities on chest CT (No.,%)		46/48 (95.8)	18/18 (100.0)	1.000
Total CT score (mean ± SD)	0–25	2 ± 2 (0–6)	6 ± 4 (2–12)	.021
CT score according to lobe (mean ± SD)	0–5			
Left upper lobe		0 ± 1 (0-2)	1 ± 1 (1-3)	
Left lower lobe		1 ± 1 (0-3)	1 ± 1 (1-5)	
Right upper lobe		0 ± 1 (0-2)	1 ± 1 (0-2)	
Right middle lobe		0 ± 1 (0-2)	1 ± 1 (0-2)	
Right lower lobe		1 ± 1 (0-2)	2 ± 1 (0-4)	

Data are presented as mean (SD, standard deviation), medians (interquartile ranges, IQR) and n/N (%).

*p* Values denoted the comparison between the survival group and death group. *p* < .05 was considered statistically significant (shown in bold).

**Table 4.** Multivariate ORs for the prediction of death in hemodialysis patients with COVID-19.

Predictor	Odds ratio	95%Confidence interval	*p*
Age	0.980	0.974–1.037	.514
Fever	1.477	1.014–1.439	.044
Dyspnea	1.146	1.026–1.875	.034
Blood leukocyte count	0.938	0.859–1.024	.155
Prothrombin time	1.033	1.003–1.065	.132
D-dimer	4.974	3.315–6.263	.007

Among the deceased hemodialysis patients, respiratory and heart failure were numerous. Severe complications observed in death group included acute respiratory distress syndrome (9/18; 50.0%), respiratory failure (12/18; 66.7%), acute cardiac injury (12/18; 66.7%), heart failure (15/18; 83.3%), and shock (6/18; 33.3%). These were significantly more frequent than in the survival group, showing their potential association with the clinical outcome. Less common complications in death group included sepsis (2/18; 11.1%), disseminated intravascular coagulation (1/18; 5.6%), acute liver injury (4/18; 22.2%), and gastrointestinal bleeding (1/18; 5.6%). Complications observed in the survival group included acute cardiac injury (4/48; 8.3%), heart failure (4/48; 8.3%), acute liver injury (1/48; 2.1%), and gastrointestinal bleeding (1/48; 2.1%).All hemodialysis patients with COVID-19 received antiviral therapy (i.e. oseltamivir, Arbidol, or Ribavirin), and 64(97.0%) patients received antibacterial treatment (i.e. moxifloxacin, cefoperazone, or azithromycin). Three (16.7%, 3/18) patients in the death group and 4(8.3%, 4/48) patients in the survival group received glucocorticoids. Only 4 patients in the death group received ICU admission, while no patient in the survival group needed to be transferred to the ICU. Four patients (22.2%, 4/18) in the death group received high-flow oxygen, and 2 (11.1%, 2/18) received noninvasive ventilation. Six elderly hemodialysis patients with COVID-19 gave up tracheal intubation and invasive ventilator treatment because their families refused. No patients in the survival group needed high-flow oxygen or ventilation treatments. More importantly, the median time from diagnosis to death in the death group was only five days (range 2 to 10). Most patients died from respiratory failure and heart failure.

## Discussion

Novel coronavirus disease is now a worldwide pandemic. This virus causes more deaths than the flu infection (10–20 times more lethal) and is unfortunately much more contagious than the flu. Thousands of people died from novel coronavirus infection [9]. Hemodialysis patients, due to their own basic diseases, compromised immune function, and long-term close contact with the hospital, become very vulnerable to novel coronavirus infection [10,11].

As the largest hemodialysis center in Wuhan, our center had 602 maintenance hemodialysis patients. The hemodialysis patients were regularly dialyzed three times a week, at least 4 h per session, with poly-sulfone membrane dialyzers. The blood flow rate was adjusted from 250 to 300 mL/min. The dialysate flow rate was fixed at 500 mL/min, and ultrafiltration rates were set according to individual needs. The single pool urea kinetic model was monitored to ensure a delivered dialysis dose of at least 1.2 per dialysis for thrice weekly sessions. The clinical data were collected from 66 hemodialysis patients with COVID-19 in our center. According to different prognosis, they were divided into the survival group and death group. Compared with the survival group, a higher incidence of fever and dyspnea was presented in the death group. Meanwhile, patients in the death group were often accompanied by higher white blood cell count, prolonged PT time, increased D-dimer. More patients in the death group showed hepatocytes and cardiomyocytes damage. Furthermore, logistic regression analysis suggested that fever, dyspnea, and elevated D-dimer level were independent risk factors for death in hemodialysis patients with COVID-19.

Similar to the research of non-dialysis patients with COVID-19 [12–14], higher white blood cell count, longer PT time, and higher D-dimer level were also found in hemodialysis patients with poor prognosis after infected with coronavirus. These phenomena might suggest that severe inflammatory damage and coagulation system activation indicated bad outcomes.

Hepatocyte damage was also observed in both hemodialysis and non-hemodialysis patients who died from COVID-19 [12,13,15]. Furthermore, in our observation, hemodialysis patients are also accompanied by cardiomyocyte damage, which is mainly manifested by elevated troponin I level. Nevertheless, only one 40-year-old male hemodialysis patient with elevated troponin I progressed to acute myocardial infarction. After treatment with anti-bacterium, antiviral, anticoagulation, and inhibition of platelet aggregation, this patient finally resolved.

In our observation, hemodialysis patients with COVID-19 showed some clinical characteristics different from the general population. First, compared with the data from 1099 patients infected with the novel coronavirus, the incidence of fever in our center is not as high as that of the general population (37.9% vs. 87.9%) [3]. Although the prevalence of fever in the death group is much higher than that in the survival group and may be related to the prognosis. Second, in another single-center case series of 138 hospitalized patients with COVID-19, it was observed that patients admitted to the intensive care unit were older and had more complications than non-ICU patients [12]. Another clinical research also suggested that older age and higher D-dimer might be potential risk factors of poor prognosis at an early stage of disease [13]. But in our observation, unlike non-dialysis patients, no significant difference in age was found between the death group and the survival group. The logistic regression analysis suggested that no correlation between the age and death risk of hemodialysis patients with COVID-19. Therefore, even young hemodialysis patients should be highly vigilant if they develop fever and dyspnea at the beginning of disease. The appearance of these symptoms may indicate an adverse outcome.

Recently, it was observed that the activation of alveolar macrophages was a characteristic lesion of lung pathological progression in severe patients [16]. Unfortunately, we did not find the changes in peripheral blood macrophages in infected hemodialysis patients (data not shown). The role of macrophages in the pathological progress of hemodialysis patients may need more observations.

Although we had taken many measures to reduce the infection rate of hemodialysis patients, such as education of staff and patients, expanding the spatial distance between patients, strengthening the disinfection of dialysis rooms, speeding up the screening of suspicious patients, and separating of infected or symptomatic and non-infected patients [17]; unfortunately, the incidence of novel coronavirus infection in our hemodialysis center was up to 11.0%(66/602), of which 18(27.3%, 18/66) hemodialysis patients died. The mortality rate of COVID-19 in our center surpassed the overall mortality rate in Wuhan (nearly 5.1%, 2574/50008). The median time from diagnosis to death in hemodialysis patients with COVID-19 was very short. In the early stage of the disease outbreak, especially from January 27 to February 6, the number of COVID-19 patients increased rapidly, but the Huoshenshan hospital and cabin hospitals were still under construction. Consequently, medical resources such as the ICU ward and medical staff, high flow nasal cannula oxygen ventilator, extracorporeal membrane oxygenation (ECMO) were in shortage at Wuhan during that time. On the other hand, some family members of elderly patients refused invasive rescue treatments according to their own wishes. Besides, some hemodialysis patients with COVID-19 quickly developed severe complications such as cardiac arrest, hypotensive shock because of viral virulence and cardiovascular co-existing orders. These patients often died before invasive rescue treatment was given. Therefore, the shortage of medical resources, the wishes of family members, and time constraints might lead to a higher mortality rate than the general population in our center. After February 15th, a large number of mobile cabin hospitals were put into use, the specialized hospitals such as Huoshenshan, Leishenshan, and Jinyintan hospital were in good operation, and ICU medical staff across the country rushed to help. These measures have effectively reduced the mortality rate of COVID-19 patients, including hemodialysis patients with COVID-19.

This study has several limitations. First, the observation time is too short to study the long-term damage and prognosis of hemodialysis patients with novel coronavirus infection. Second, patients were sometimes transferred late in their illness to designated hospitals. Lack of invasive mechanical ventilation and extracorporeal membrane oxygenation (ECMO) might have also contributed to the poor clinical outcomes in some patients. Third, by excluding hemodialysis patients with negative RT-PCR results but suspicious chest CT scans, the case fatality ratio in our study might be higher than the true mortality in hemodialysis patients with COVID-19.

## Conclusions

COVID-19 poses great challenges to patients on maintenance hemodialysis. Clinicians should be aware of the risk of death in hemodialysis patients with COVID-19. The potential risk factors of fever, dyspnea, and elevated D-dimer could help clinicians to identify hemodialysis patients with poor prognosis at an early stage.
